# A Group of Distinguished Physicians and Surgeons of Chicago

**Published:** 1904-08

**Authors:** 


### Bibliography.

A Group of Distinguished Physicians and Surgeons of Chicago. Compiled by F. M. Sperry. Illustrated. J. H. Beers & Co., Chicago, Ill., 1904.

This is a handsome volume of two hundred and forty pages, and worthy of the men and women who are embalmed within its covers. While books of this character abound, from "Who's Who?" to smaller specimens of the personal glorification order, this book seems to be free from the usual criticism, and is really what its title indicates,—a history of the prominent physicians of Chicago.

It is eminently proper that Dr. N. S. Davis should occupy the frontispiece. While Chicago has a due proportion of men with national and international reputations, no one of these better represents the broad, liberal medical thought of the day than Dr. Davis. Dentistry holds him in grateful remembrance for his many kind words and deeds in its behalf. Not that our profession needs special aid and encouragement, but that out of the mists of medical prejudice that have beclouded the mental horizon of that profession since the days of Harris and Hayden there could be found one man who could see beyond this into the clearer life and hygienic value of the work of dentistry and its intimate relation to the healing art. While dentistry is perfectly able to maintain its independent position, forced upon it by Dr. Davis's predecessors, it nevertheless feels that the appreciation of such a man means added honor to its work.

Among the illustrations of this book are several of prominent women in medical practice in Chicago. The writer has not to go back very far in medical history to recall a period when the medical men of that day would have felt themselves humiliated to have been embalmed in a volume with medical women. The fact that this generation has outgrown the narrow professional bigotry of the past is an encouragement to believe that that profession is steadily outgrowing the swaddling clothes of medical infancy.

Dentistry finds an honorable place in this volume. Very excellent portraitures of Eugene S. Talbot, M.D., D.D.S., and Truman W. Brophy, M.D., D.D.S., LL.D., are among its illustrations.

It is interesting to read in the history of Dr. Talbot traces of the beginning of the active life of this renowned thinker, investigator, and voluminous contributor to the scientific side of medicine and dentistry. It is well sometimes to recur to these personal histories of distinguished men, as they furnish valuable lessons and are oftentimes corrective of narrow conceptions and conclusions. Dr. Talbot was born in Massachusetts, and received a public school education, " followed by academic training at Stoughtonham Institute until the age of sixteen years." He apprenticed himself to the South Boston Locomotive Works, and was trained to work on marine engines. He became a master mechanic at nineteen, and was, subsequently, foreman at the Pennsylvania Railroad Repair Shops. He subsequently graduated in dentistry and medicine, the former at the Pennsylvania College of Dental Surgery, and the latter at Rush Medical College, Chicago.

In the very strenuous demand for higher preliminary education, it may be well to recur occasionally to the fact that the future of a young man does not depend so much on the degree secured as upon the determination to build upon that which has been obtained. The lesson of Dr. Talbot's life is, therefore, instructive in this direction, and should be an encouragement and incentive to higher work throughout the graduated body in dentistry of recent years.

Dr. Brophy's career is of very similar character to Dr. Talbot's. Like the latter he received his education in the public school and academy at Elgin, Ill. His professional education was carried on in the Pennsylvania College of Dental Surgery and Rush Medical College of Chicago.

His achievements as a dental educator and surgeon are well known. His one contribution, among many, to oral surgery, that of the radical cure of cleft palate, known as the "Brophy operation," has made his name known throughout the surgical world.

These facts are recalled here that the young men entering dentistry may not be unmindful that energy and persistent cultivation of the mental forces will insure a place in the world's regard, and this can be accomplished in no other way. It is through just such efforts that dentistry has been recognized at its true worth,—one of the most important professions of modern life and superior to some in its hygienic relations and its possibilities in the prolongation of human life. What is of greater importance, however, is the continuance of the mental powers to an indefinite period in the life of the individual through its advanced practice in the treatment of the oral cavity, the vestibule of the entire organism.

